# Different Stimulation Frequencies Alter Synchronous Fluctuations in Motor Evoked Potential Amplitude of Intrinsic Hand Muscles—a TMS Study

**DOI:** 10.3389/fnhum.2016.00100

**Published:** 2016-03-07

**Authors:** Martin V. Sale, Nigel C. Rogasch, Michael A. Nordstrom

**Affiliations:** ^1^Queensland Brain Institute, The University of QueenslandSt Lucia, QLD, Australia; ^2^Brain and Mental Health Laboratory, School of Psychological Science and Monash Biomedical Imaging, Monash Institute of Cognitive and Clinical Neuroscience, Monash UniversityMelbourne, VIC, Australia; ^3^Discipline of Physiology, School of Medical Sciences, The University of AdelaideAdelaide, SA, Australia

**Keywords:** transcranial magnetic stimulation, cortical oscillations, motor-evoked potential, motor cortex, first dorsal interosseous, abductor pollicis brevis, abductor digiti minimi

## Abstract

The amplitude of motor-evoked potentials (MEPs) elicited with transcranial magnetic stimulation (TMS) varies from trial-to-trial. Synchronous oscillations in cortical neuronal excitability contribute to this variability, however it is not known how different frequencies of stimulation influence MEP variability, and whether these oscillations are rhythmic or aperiodic. We stimulated the motor cortex with TMS at different regular (i.e., rhythmic) rates, and compared this with pseudo-random (aperiodic) timing. In 18 subjects, TMS was applied at three regular frequencies (0.05 Hz, 0.2 Hz, 1 Hz) and one aperiodic frequency (mean 0.2 Hz). MEPs (*n* = 50) were recorded from three intrinsic hand muscles of the left hand with different functional and anatomical relations. MEP amplitude correlation was highest for the functionally related muscle pair, less for the anatomically related muscle pair and least for the functionally- and anatomically-unrelated muscle pair. MEP correlations were greatest with 1 Hz, and least for stimulation at 0.05 Hz. Corticospinal neuron synchrony is higher with shorter TMS intervals. Further, corticospinal neuron synchrony is similar irrespective of whether the stimulation is periodic or aperiodic. These findings suggest TMS frequency is a crucial consideration for studies using TMS to probe correlated activity between muscle pairs.

## Introduction

The amplitude of the motor-evoked potential (MEP) evoked with transcranial magnetic stimulation (TMS) varies from one trial to the next. The MEP fluctuations are due, at least in part, to changes in corticospinal neuron excitability (Burke et al., 1995; Ellaway et al., 1998; Funase et al., 1999), reflecting a moment-to-moment fluctuation in the balance of excitatory and inhibitory neural inputs acting on the corticospinal neurons. There is widespread coupling of corticospinal neuron excitability both within, and between, the hand areas of motor cortex (Pearce et al., 2005). The precise mechanism and function of this neural coupling is not clear at present, but widespread synchronous fluctuations in excitability may assist the nervous system to dynamically link groups of neurons in the brain for particular functional tasks (Fries et al., 2007). Synchronous discharge of two inputs to a neuron is more effective at influencing that neuron’s discharge than two uncorrelated inputs of equivalent strength. Neural synchrony is found throughout the central nervous system (CNS), and a pre-eminent theory for the function of this synchrony in sensory systems is that it could serve as the basis for perceptual binding (Eckhorn et al., 1988; Singer and Gray, 1995). In the motor system, synchrony could serve to dynamically link together networks of cells into a functional cell assembly for the performance of motor tasks (Farmer, 1998; Baker et al., 1999; Brown, 2000; Brown and Marsden, 2001; Jackson et al., 2003; Sanes and Truccolo, 2003). This dynamic cortical synchrony could determine, for example, the combination of muscles required for a task, which would vary depending on the requirements of the task (and external conditions).

Moment-to-moment fluctuations in MEP amplitude between muscle pairs appear to be somatotopically organized (Pearce et al., 2005). The correlation in MEPs is greater for intrinsic hand muscles compared to the correlation between an intrinsic and extrinsic hand muscle of the same limb. However, whether there are differences in correlations between intrinsic hand muscles that have different functional and anatomical relationships is unknown. In the present study, we measured fluctuations in three separate hand muscle pairs (first dorsal interosseous, FDI; abductor pollicis brevis, APB; abductor digiti minimi, ADM) that share different functional and anatomical relationships. We hypothesized that if MEP fluctuations have an important role in functional binding, then fluctuations would be greater for functionally related muscle pairs FDI and APB.

Synchronous discharges from neural assemblies can be oscillatory (rhythmic) or randomly timed (aperiodic). The motor system exhibits rhythmic oscillations in neural activity over a range of frequencies (~1–100 Hz), and some of these have been implicated in binding (Farmer, 1998; Brown, 2000; Brown and Marsden, 2001). Several studies have observed inverse relationships between trial-to-trial variation in MEP amplitude and spontaneous fluctuations in pre-TMS oscillatory power in both alpha [8–12 Hz] (Zarkowski et al., 2006; Sauseng et al., 2009) and beta [15–30 Hz] (Mäki and Ilmoniemi, 2010; Keil et al., 2014; Schulz et al., 2014) bands. In addition, synchronized fluctuations in MEP amplitude between contralateral muscle pairs have been linked with gamma oscillations [40 Hz] (Funk and Epstein, 2004). These spontaneous fluctuations in oscillatory power between 1 and 40 Hz are further nested within slow (0.1–1 Hz) and infra-slow (0.01–0.1 Hz) oscillations (Monto et al., 2008). These slow oscillations are thought to represent drifts in membrane potentials (Palva and Palva, 2012) that could influence MEP variability and synchronization. Indeed, a recent study demonstrated that MEP amplitude was differentially modulated by up- and down-states of slow oscillations (0.16–2 Hz) induced by the early stages of sleep (Bergmann et al., 2012). These findings suggest that the amplitude and variability of TMS-evoked MEPs can be influenced by endogenous cortical rhythms.

In the present study, we investigated oscillations in motor cortical excitability by stimulation of the motor cortex with TMS at three regular slow and infra-slow frequencies, and compared these with TMS delivered with pseudo-random timing. If the mechanism producing the common fluctuations in excitability is rhythmic, we expect to see a weaker correlation in the size of responses evoked in different muscles when pseudo-random timing is used for the TMS. If a particular frequency of TMS is more effective at producing synchronous fluctuations in MEP size in hand muscles that would be further evidence for a rhythmic process underlying the synchronous fluctuations in corticospinal neuron excitability. The results of the study will improve our understanding of the mechanisms operating in human motor cortex that dynamically link the output delivered to different muscle groups.

## Materials and Methods

Eighteen neurologically normal right-handed subjects (11 female; aged 20–48 years) participated in the study. Each subject was tested at approximately the same time of day (early afternoon, ~2 pm) to make any circadian effects on motor cortex excitability as uniform across subjects as possible (Sale et al., 2007, 2008, 2010). All subjects gave written informed consent prior to participation in the study, which was approved by the University of Adelaide Human Research Ethics Committee.

### Stimulation and EMG Recording

Subjects were seated in a dental chair with their left arm resting comfortably on their lap. Surface electromyography (EMG) was obtained using bipolar Ag-AgCl electrodes placed 2 cm apart over *abductor pollicis brevis* (APB), *first dorsal interosseous* (FDI) and *abductor digiti minimi* (ADM) muscles of the left hand in a belly-tendon montage. EMG signals were amplified (1000×), bandpass filtered (20–1000 Hz) and digitized (2 kHz/channel) via a CED 1401 interface, and stored on computer for offline analysis. The EMG signals of all muscles were displayed for the subject on oscilloscopes to assist them in maintaining EMG silence. Trials containing voluntary activity in any muscle group were excluded from analysis.

### Transcranial Magnetic Stimulation

A single-pulse TMS was delivered by a Magstim 200 magnetic stimulator (Magstim, Dyfed, UK) through a 70 mm figure-of-eight coil. The coil was positioned at a location that optimally evoked MEPs in left FDI. The handle of the coil pointed posteriorly, so as to induce current flow in a posterior-anterior (PA) direction in the right motor cortex. This site was marked with a pen, and the position of the coil relative to the site was continually checked throughout the experiment to avoid coil displacement.

The threshold for evoking MEPs in relaxed left FDI was established. Resting motor threshold (RMT) was defined as the minimum TMS intensity required to evoke MEPs of an amplitude >50 μV peak-to-peak in 5 out of 10 consecutive trials. The TMS intensity was expressed as a percentage of the maximum stimulator output (%MSO). Following this, a test TMS intensity was determined. This was defined as the TMS intensity sufficient to evoke MEPs with a peak-to-peak amplitude in the range of 0.5–1.0 mV in relaxed FDI, which also consistently produced MEPs in the other two muscles.

### Stimulation Frequencies

Blocks of four different TMS frequencies in the range of slow and infra-slow oscillations were used to determine whether TMS frequency influenced the correlation of MEP amplitude between different muscle pairs. TMS frequencies used were: 0.05 Hz, 0.2 Hz, 1 Hz, and an aperiodic pattern with variable interstimulus intervals and a mean rate of 0.2 Hz. In the aperiodic stimulation condition, TMS was delivered at a mean rate of one stimulus every 5 s (0.2 Hz), with the stimulation rate pseudorandomly varied between the upper limit of one stimulus every 2.5 s (0.4 Hz) and a lower limit of one stimulus every 7.5 s (0.13 Hz). The distribution of the stimulation frequencies within these limits was uniformly distributed. The order in which the subject received the different stimulation protocols was randomized. For each protocol, 50 TMS were delivered. To avoid the induction of neuroplastic effects with 1 Hz stimulation (Chen et al., 1997), this block was divided into 5 blocks of 10 (1 Hz) TMS pulses, with a break of several minutes between each block. During each break period, the subject was instructed to stay as still as possible, with hand muscles relaxed.

To ensure that any differences in MEP amplitude within each block were not due to neuroplastic changes resulting from the frequency of stimulation, a paired *t*-test was performed on mean MEP amplitudes of each muscle from the first 25 stimuli in each block and compared to the MEP amplitudes from the last 25 stimuli in that block for all stimulus frequencies (Pearce et al., 2005). There were no significant changes in MEP amplitudes across the first and second blocks of stimuli for any muscles and stimulus frequencies.

### Statistical Analysis

All muscle pairs were subjected to linear regression analysis and further statistical analysis of the correlation coefficient. A two-way repeated measures analysis of variance (ANOVA) was used to assess the effect of STIMULATION PROTOCOL (four levels: 0.05 Hz, 0.2 Hz [regular], 1 Hz, mean 0.2 Hz [aperiodic]) and MUSCLE PAIR (three levels: APB-FDI, FDI-ADM, APB-ADM) on the extent of correlated changes (*r^2^*) in MEP amplitude. The muscle pairings have different functional and anatomical relationships. APB and FDI are functionally related, and are used in many precision grip tasks, but are innervated by different nerves (median and ulnar nerves, respectively). FDI and ADM are commonly innervated by the ulnar nerve but are functionally unrelated, whereas APB and ADM are both functionally and anatomically unrelated.

To assess whether TMS pulses given at different frequencies interacted with the amplitude of MEPs evoked with subsequent TMS pulses, auto-correlations were performed on MEP amplitudes within each muscle at lags of 1, 2 and 3 pulses using the *autocorr* function in Matlab (r2105a, Mathworks). Separate two-way repeated measures ANOVAs assessed the effect of MUSCLE (three levels: APB, FDI, ADM) and STIMULATION PROTOCOL (four levels) on the auto-correlation coefficient at each lag.

Separate two-way repeated measures ANOVAs assessed the effect of MUSCLE (three levels: APB, FDI, ADM) and STIMULATION PROTOCOL (four levels) on MEP amplitude and the coefficient of variation (CV) of MEP amplitude during each trial.

Data are reported as means ± SEM, and *P* < 0.05 was considered significant. Significant effects were followed up with two-tailed *t* tests using the Bonferroni correction for multiple comparisons (the correction has been applied to the reported *P* value, so that *P* < 0.05 indicates significance). The Greenhouse-Geisser correction was used for violations of sphericity.

## Results

All subjects completed the experiments, and no adverse effects were noted.

### RMT and TMS Test Intensity

The mean RMT for left FDI was 57.4 ± 1.8% MSO, and the mean TMS intensity of the test stimulus was 70.7 ± 2.6% MSO.

### MEP Variability

**Figure 1:**
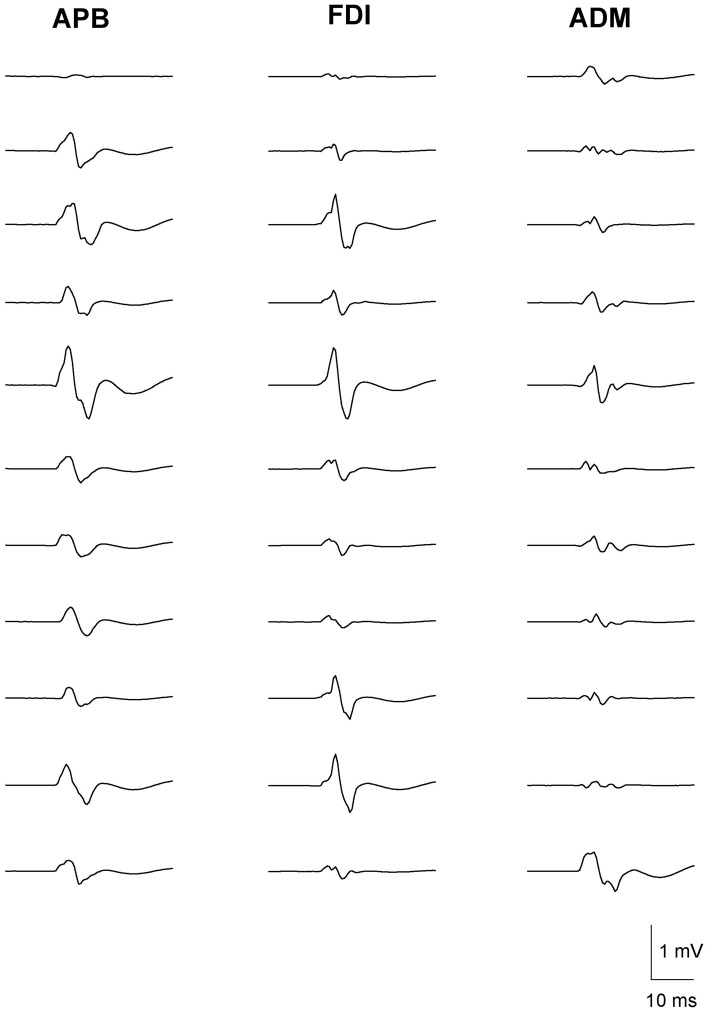
**Fluctuation in motor-evoked potential (MEP) amplitudes recorded from left abductor pollicis brevis (APB) (left column), first dorsal interosseous (FDI) (middle column) and abductor digiti minimi (ADM) (right column) in a representative subject.** Data are from 11 consecutive TMS trials, with a stimulation frequency of 0.2 Hz. MEP amplitudes varied considerably during the trials. The greatest correlation in MEP amplitude fluctuation is observed between the functionally-related muscle pair of APB-FDI. Less correlation in MEP amplitude fluctuation was observed between the anatomically related muscle pair of FDI-ADM. Least correlation in MEP amplitude fluctuation was seen between the functionally- and anatomically-unrelated muscle pair of APB-ADM. The correlation coefficients of the corresponding trials (*n* = 50) were: APB-FDI *r^2^* = 0.67; FDI-ADM *r^2^* = 0.58; APB-ADM *r^2^* = 0.31.

The variability in MEP amplitudes from one representative subject with 11 consecutive stimuli in the three muscle groups is shown in Figure 1. The stimuli were delivered at a frequency of 0.2 Hz. There was considerable variability in the amplitude of MEPs in successive trials within the same muscle. However, the trial-to-trial variability of MEPs between different muscles showed a greater correlation in the amplitude of MEPs between APB and FDI, than APB and ADM or FDI and ADM.

**Figure 2:**
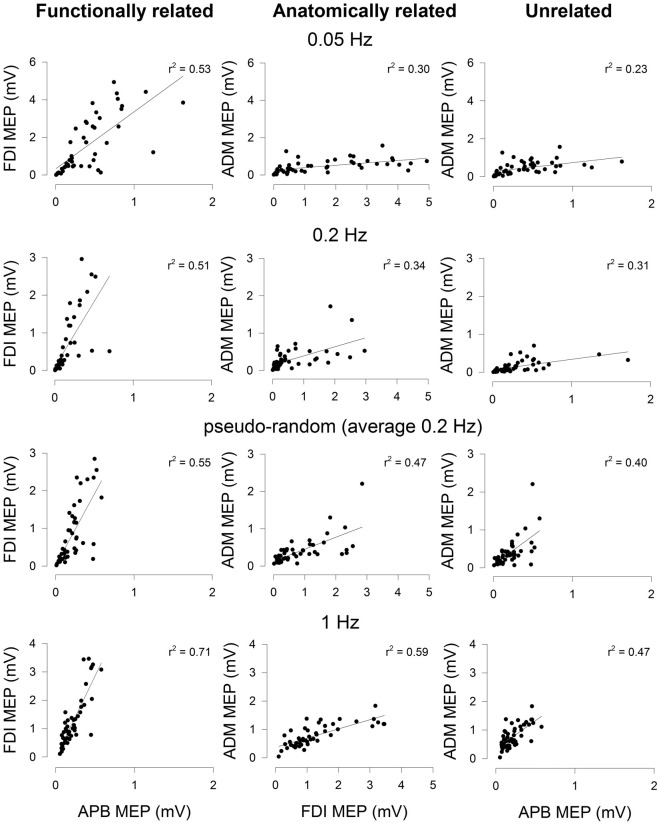
**Correlation of MEP amplitudes between a functionally-related muscle pair (APB-FDI; left column), an anatomically related muscle pair (FDI-ADM; middle column) and a functionally and anatomically unrelated muscle pair (APB-ADM; right column).** Data are from one representative subject and were derived from trials of 50 consecutive TMS stimuli delivered focally to the right hemisphere at four different stimulation protocols (0.05 Hz; top row, 0.2 Hz; row second from top, aperiodic frequency (average 0.2 Hz); row third from top, 1 Hz; bottom (row). The correlation of MEP amplitudes was higher in the functionally related muscle pair than either the anatomically related or unrelated muscle pairs. The correlation of MEP amplitudes in the anatomically related muscle pair was higher than the unrelated muscle pair. Higher frequency stimulation tended to result in MEP amplitudes that were more correlated between muscle pairs, irrespective of the type of muscle pairing. Significant linear regression lines and *r^2^* values are shown (*P* < 0.05).

The data from a representative subject (different from Figure 1) showing the correlation in MEP amplitude for all muscle pairs assessed with the different stimulation protocols is shown in Figure 2. The correlation of MEPs for all muscle pairs was greater at higher stimulation frequencies. At each stimulation protocol, the correlation of MEPs was greater between FDI and APB muscles (left-most column).

**Figure 3:**
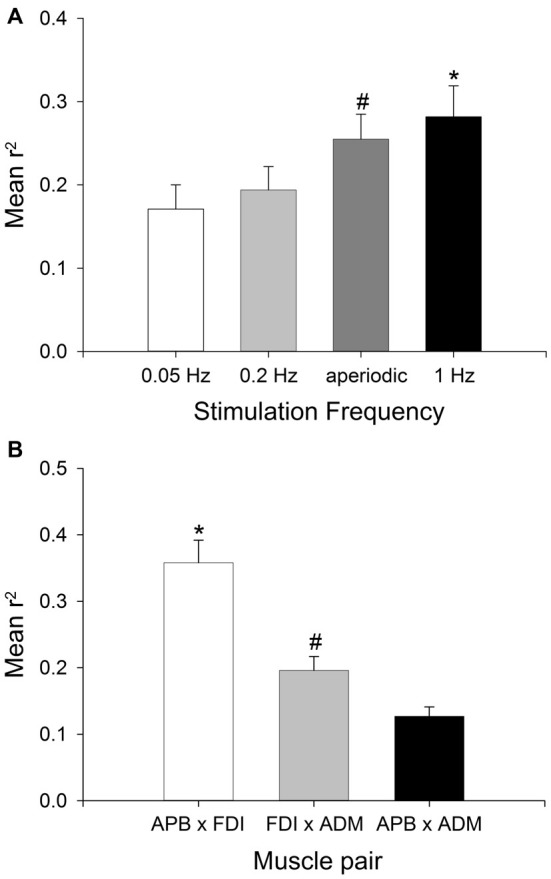
**Effect of stimulation frequency and muscle pairings on the strength of MEP correlations.** Group (mean ± SEM) data showing the effect of TMS stimulation frequency **(A)**, and muscle pairing **(B)** on the strength of correlation of MEP size fluctuations in 18 subjects. Mean *r^2^* was significantly higher with a stimulation frequency of 1 Hz compared to stimulation at 0.05 Hz or 0.2 Hz (*P < 0.05), and significantly higher with aperiodic stimulation than 0.05 Hz stimulation (^#^*P* < 0.05). Mean *r^2^* was significantly higher in the APB-FDI muscle pairing compared to both FDI-ADM and APB-ADM muscle pairings (*P < 0.001), and the mean *r^2^* for the FDI-ADM muscle pairing was significantly greater than for the APB-ADM muscle pairing (^#^*P* < 0.05).

The group data for MEP amplitude correlation across three muscle pairs and using four stimulation protocols are shown in Figure 3. The ANOVA revealed a significant main effect of STIMULATION PROTOCOL (*F*_(3,102)_ = 3.53, *P* < 0.05, $\eta_{p}^{2}$ = 0.172) and no significant interaction between STIMULATION PROTOCOL and MUSCLE PAIR (*F*_(6,102)_ = 1.34, *P* > 0.05, $\eta_{p}^{2}$ = 0.073). MEP correlations were largest with the highest stimulation frequency (1 Hz; mean *r^2^* = 0.273 ± 0.035), and lowest with the slowest stimulation frequency (0.05 Hz; mean *r^2^* = 0.191 ± 0.032). Subsequent pairwise comparisons revealed that MEP amplitude correlation was significantly greater with stimulation at 1 Hz than a stimulation frequency of either 0.05 Hz (*t* = 2.88, *P* < 0.05) or 0.2 Hz (*t* = 3.09, *P* < 0.05). MEP amplitude correlation was significantly greater with aperiodic stimulation than stimulation at 0.05 Hz (*t* = 2.87, *P* < 0.05; Figure 3A). There was no significant difference between 0.2 Hz stimulation and aperiodic stimulation (mean 0.2 Hz). There was also a significant main effect of MUSCLE PAIR (*F*_(2,102)_ = 12.66, *P* < 0.05, $\eta_{p}^{2}$ = 0.427). Subsequent pairwise comparisons revealed that the functionally related muscle pair of APB-FDI had a significantly greater correlation in MEP amplitudes than either APB-ADM (*t* = 7.92, *P* < 0.05) or FDI-ADM (*t* = 4.55, *P* < 0.05; Figure 3B). The commonly innervated muscle pair of FDI-ADM had a significantly greater correlation in MEP amplitudes than the functionally and anatomically unrelated muscle pair of APB-ADM (*t* = 5.45, *P* < 0.05; Figure 3B).

The potential influence of a TMS pulse on the amplitude of MEPs evoked with subsequent TMS pulses was also investigated, to determine whether TMS had a temporal influence on MEP amplitudes, and whether this was affected by stimulation frequency. Autocorrelation analyses were performed for TMS pulses and their subsequent effects on the amplitude of the following three MEPs (lag 1, 2 and 3). At lag 1 (i.e., whether the amplitude of an MEP influences the amplitude of the MEP evoked with the next TMS pulse in the same muscle), the repeated measures ANOVA revealed a significant effect of STIMULATION PROTOCOL (*F*_(3,102)_ = 12.39, *P* < 0.05, $\eta_{p}^{2}$ = 0.694). Subsequent pairwise comparisons revealed that MEP autocorrelation at lag 1 was significantly greater with stimulation at 1 Hz (0.31 ± 0.03) than a stimulation frequency of 0.2 Hz (*t* = 4.29, *P* < 0.05; 0.16 ± 0.02), 0.05 Hz (*t* = 2.80, *P* < 0.05; 0.07 ± 0.02) and aperiodic stimulation (*t* = 4.12, *P* < 0.05; 0.16 ± 0.02). MEP autocorrelation at lag 1 was significantly greater with stimulation at 0.2 Hz than a stimulation frequency of 0.05 Hz (*t* = 2.80, *P* < 0.05), and aperiodic stimulation was also greater than 0.05 Hz stimulation (*t* = 2.99, *P* < 0.05). There was no significant effect of MUSCLE (*F*_(2,102)_ = 2.79, *P* > 0.05, $\eta_{p}^{2}$ = 0.191), nor was there a significant STIMULATION PROTOCOL × MUSCLE interaction (*F*_(6,102)_ = 1.39, *P* > 0.05, $\eta_{p}^{2}$ = 0.550). At lag 2 (i.e., whether the amplitude of an MEP influences the amplitude of the MEP evoked two TMS pulses later in the same muscle), the repeated measures ANOVA revealed a significant effect of STIMULATION PROTOCOL (*F*_(3,102)_ = 9.65, *P* < 0.05, $\eta_{p}^{2}$ = 0.362). Subsequent pairwise comparisons revealed that MEP autocorrelation at lag 2 was significantly greater with stimulation at 1 Hz (0.23 ± 0.03) than a stimulation frequency of 0.2 Hz (*t* = 4.30, *P* < 0.05; 0.08 ± 0.02), 0.05 Hz (*t* = 6.34, *P* < 0.05; 0.01 ± 0.02) and aperiodic stimulation (*t* = 4.54, *P* < 0.05; 0.08 ± 0.02). There was no significant effect of MUSCLE (*F*_(2,102)_ = 0.43, *P* > 0.05, $\eta_{p}^{2}$ = 0.025), nor was there a significant STIMULATION PROTOCOL × MUSCLE interaction (*F*_(6,102)_ = 1.10, *P* > 0.05, $\eta_{p}^{2}$ = 0.061). At lag 3 (i.e., whether the amplitude of an MEP influences the amplitude of the MEP evoked three TMS pulses later in the same muscle), the repeated measures ANOVA revealed a significant effect of STIMULATION PROTOCOL (*F*_(3,102)_ = 5.59, *P* < 0.05, $\eta_{p}^{2}$ = 0.247). Subsequent pairwise comparisons revealed that MEP autocorrelation at lag 3 was significantly greater with stimulation at 1 Hz (0.17 ± 0.03) than a stimulation frequency of 0.2 Hz (*t* = 4.80, *P* < 0.05; 0.10 ± 0.02) or 0.05 Hz (*t* = 2.60, *P* < 0.05; 0.03 ± 0.02). There was no significant effect of MUSCLE (*F*_(2,102)_ = 0.50, *P* > 0.05, $\eta_{p}^{2}$ = 0.029), nor was there a significant STIMULATION PROTOCOL × MUSCLE interaction (*F*_(6,102)_ = 2.64, *P* > 0.05, $\eta_{p}^{2}$ = 0.134). These results suggest that a TMS pulse can have an important effect on the amplitude of MEPs evoked by subsequent TMS pulses. This effect becomes progressively less prominent as the lag between MEPs increases, and is largest for the highest stimulation frequency (i.e., 1 Hz).

When comparing MEP amplitudes, the ANOVA revealed no significant main effect of MUSCLE (*F*_(2,102)_ = 1.51, *P* > 0.05, $\eta_{p}^{2}$ = 0.081) on MEP amplitude, indicating that the size of the MEPs evoked with the test TMS was equivalent between muscles. There was also no significant main effect of STIMULATION PROTOCOL (*F*_(3,102)_ = 0.22, *P* > 0.05, $\eta_{p}^{2}$ = 0.013) on MEP amplitude, nor a significant STIMULATION PROTOCOL × MUSCLE interaction (*F*_(6,102)_ = 0.55, *P* > 0.05, $\eta_{p}^{2}$ = 0.031). This is important as it shows no overall difference in MEP size related to stimulation protocol (e.g., due to plasticity effects, different patterns of TMS activation of the targeted cortical neurons, or some other factor affecting MEP amplitudes that might affect the correlations).

There was no significant main effect of MUSCLE (*F*_(2,102)_ = 2.02, *P* > 0.05, $\eta_{p}^{2}$ = 0.106) on CV of MEP amplitude, indicating that the variability of MEPs evoked during a trial was equivalent across muscles. There was no significant main effect of STIMULATION PROTOCOL (*F*_(3,102)_ = 0.325, *P* > 0.05, $\eta_{p}^{2}$ = 0.019) on CV of MEP amplitude, nor a significant STIMULATION PROTOCOL × MUSCLE interaction (*F*_(6,102)_ = 0.23, *P* > 0.05, $\eta_{p}^{2}$ = 0.013).

## Discussion

The present study sought to investigate whether the trial-to-trial variability in MEP amplitudes recorded from three intrinsic hand muscles of the left hand evoked by suprathreshold TMS to M1 was influenced by the temporal pattern of stimulation, and whether this was influenced by the functional and anatomical relation between muscle pairs. We found that the degree of correlation of MEP amplitude fluctuations across the three muscle pairs was dependent on stimulation frequency. The correlation in MEP amplitude fluctuations tended to increase with increasing stimulation frequency, with the lowest correlation reported with stimulation at 0.05 Hz, and the highest at 1 Hz. Across all stimulation frequencies, the correlation of MEP amplitude fluctuations was higher between the functionally-related muscle pair (APB-FDI) than between the anatomically-related muscle pair (FDI-ADM) and least between the functionally- and anatomically-unrelated muscle pair (APB-ADM).

### Stimulation Frequency Modulates the Strength of MEP Amplitude Correlations

We have shown that the correlation of MEP amplitudes between intrinsic muscles of the left hand evoked with TMS is influenced by the frequency of stimulation. MEP correlation between hand muscles was highest with 1 Hz stimulation, and least with 0.05 Hz stimulation. The non-invasive nature of the experimental design does not allow us to provide any conclusive information on the mechanism by which this might occur, but prior research suggests some potential interpretations. If repeated TMS pulses are delivered at a frequency corresponding to the existing endogenous cortical rhythm in the targeted local cortical network (e.g., at the individual’s alpha frequency), the power of that endogenous cortical rhythm can be enhanced, and can outlast the period of stimulation (Thut et al., 2011). Thus, an increase in MEP correlations with 1 Hz stimulation might reflect entrainment of endogenous 1 Hz oscillations on inputs to the targeted corticospinal neurons. In support of this interpretation, Bergmann and colleagues demonstrated that MEP amplitudes were differentially modulated by up- and down-states of slow oscillations (0.16–2 Hz) in the early stages of sleep (Bergmann et al., 2012). However, that study used EEG to trigger the TMS pulse at specific phases of the slow oscillations, whereas we gave the stimulation periodically without accounting for phase. Alternatively, a large body of evidence now exists demonstrating that a single TMS pulse to M1 resets all neuronal oscillations of the target neurons, bringing them transiently into synchrony (for review see Rogasch and Fitzgerald, 2013). Although this synchronization has only been measured at higher frequencies (>1 Hz) and over short time periods (<500 ms), it is conceivable that slower oscillations could also be reset. For instance, hemodynamic changes measured with near-infrared spectroscopy peak approximately 8 s following a single TMS pulse. At 1 Hz stimulation frequencies, the changes in blood flow summate and persist throughout stimulation, suggesting a prolonged effect of TMS on neural activity (Thomson et al., 2011, 2012). As the synchronizing effect of a single TMS pulse is likely to dissipate over time (Thut et al., 2011), this could also help explain the graded decrease in MEP correlation strength with reduced stimulation frequency, and the progressive reduction in autocorrelation in MEP amplitudes within the same muscle with increasing temporal lag. Further support for this mechanism (i.e., the single TMS pulse resets all endogenous cortical oscillations, but with a limited half-life) is provided by the comparison of results for the periodic vs. aperiodic stimulation. Here, we found that there was no significant difference in MEP correlations between these two conditions. If the periodic stimulation was entraining an endogenous 0.2 Hz oscillation, we would have expected this condition to produce greater MEP correlation compared to the aperiodic condition. Further studies using combined TMS-EEG are required to study the resetting effects of TMS on slower oscillations.

The present study has implications for TMS-related research in which correlations of MEP amplitudes between different muscles are used as an indirect measure of common descending activity and functional connectivity. Our findings show that the interpretation of such results needs to consider the influence of stimulus frequency. Specifically, when stimulus frequency is increased, synchronized fluctuations in MEP amplitudes are greater. Thus, to avoid “contaminating” the data by introducing an artifact related to high stimulus intensities, our findings suggest that researchers should stimulate at frequencies slower than 0.2 Hz.

The present study did not investigate stimulation frequencies faster than 1 Hz. Repetitive TMS (rTMS) at stimulation frequencies at or greater than 1 Hz has been shown to induce robust plastic changes in target neurons (Chen et al., 1997). With a stimulation frequency of 1 Hz, Chen et al. (1997) reported a ~20% reduction in MEP amplitude following 810 rTMS pulses. This change in MEP amplitude is thought to reflect long term depression-like changes in synaptic efficacy. Although it is unlikely that the 50 pulses used in the present study would have been sufficient to induce such changes in synaptic efficacy, induction of plastic changes with TMS would have confounded the present results. As such, we restricted our assessment of MEP correlations to stimulation frequencies of 1 Hz or lower. However, in order to ensure that the 1 Hz stimulation did not induce plastic changes in the stimulated neurons, the 1 Hz trials were divided into two epochs, with a break in between. If plastic changes were induced within the target neurons it would be expected that the size of the MEP evoked at the start of a trial would be different from the size evoked at the end. We excluded any trials in which there was a significant difference in amplitude of MEPs evoked from the first half of the trial and those evoked from the second half of the trial. Furthermore, there was no significant difference in overall mean MEP amplitude for the four stimulation protocols. Therefore, it is unlikely that the high correlation of MEPs from all muscle pairs recorded with a 1 Hz stimulation frequency reflects plastic changes in synaptic efficacy induced with the TMS.

### MEP Amplitude Correlations are Strongest in Functionally Related Muscle Pairs

Several previous studies have investigated the moment-to-moment fluctuation in MEP amplitudes evoked in hand muscles. These studies have probed MEP size fluctuations between muscles of the same and opposite limbs, both at rest and during voluntary activation (Schieppati et al., 1996; Ellaway et al., 1998; Ho et al., 1998; Pearce et al., 2005). Although voluntary activation has been shown to reduce the strength of correlation between muscles of the same limb (Pearce et al., 2005), there is general agreement that widespread coupling in the excitability of corticospinal neurons controlling muscles of the same limb is present in humans. The results of the present study are in agreement with these findings, and demonstrate that the strength of correlation between MEP amplitude fluctuations is greatest for the functionally related muscle pair (APB-FDI), and least for the functionally and anatomically unrelated muscle pair (APB-ADM). Branched-axon corticomotoneuronal (CM) projections, which activate motoneuron pools of multiple synergistic muscles of the same hand, are likely to contribute to these MEP correlations (Fetz and Cheney, 1980). However, interhemispheric coupling of corticospinal excitability is also present, although to a lesser extent than that reported within a hemisphere for projections to muscles of the same limb (Ellaway et al., 1998; Pearce et al., 2005). This interhemispheric coupling cannot be attributed to CM cell projections, as they do not bilaterally innervate motoneuron pools of hand muscles in normal subjects (Carr et al., 1993). This coupling is also likely to contribute to the correlation of MEP amplitudes recorded from muscles of the same limb.

Although we have demonstrated a stronger correlation between MEP amplitude fluctuations in functionally related muscle pairs, we cannot exclude the possibility of EMG cross-talk between muscles influencing this result. However, for several reasons, we believe that although cross-talk may contribute to the degree of correlation between different muscle pairings, the contribution of cross-talk is relatively low. First, Kilner et al. (2002) compared cross-talk between APB, FDI and ADM (and other non-intrinsic hand muscles), and showed that cross-talk between the intrinsic hand muscles was negligible when calculating coherence estimates. Second, the signal resulting from cross talk is between 4–20 times smaller than the signal in the target muscle [APB-ADM] (Selvanayagam et al., 2012). Third, the correlation co-efficients observed between ipsilateral muscles in the current experiment (*r^2^* = 0.2–0.35) are comparable with those observed between contralateral homologous muscles which are not affected by EMG cross-talk (Pearce et al., 2005). Taken together, these findings suggest that cross-talk is unlikely to have a significant influence on the results reported in the present article.

## Conclusion

In summary, we found that MEP size fluctuations in intrinsic muscles of the left hand evoked with TMS to right M1 are influenced by stimulation frequency. Higher stimulation frequencies were associated with greater correlation in MEP amplitudes between muscle pairs. We propose that this may be due to temporary resetting of target neuron oscillatory activity by the TMS. Further, stimulation at regular intervals (e.g., 0.2 Hz in the present study) is not associated with a higher level of MEP correlation between muscle pairs compared with irregular, aperiodic stimulation (e.g., average 0.2 Hz in the present study). Irrespective of stimulation frequency of TMS, the correlation of MEP amplitudes is greatest for functionally related muscle pairings, and least for anatomically and functionally unrelated muscle pairs. These findings have important implications for studies that use repeated pulses of TMS to quantify changes in cortical excitability between different muscles. The results of the present study suggest that correlations in MEP amplitudes between muscle pairs will be inflated at high stimulation frequencies. Thus, stimulation frequencies ≤0.2 Hz should be used to avoid the TMS pulses interacting with endogenous cortical oscillatory rhythms.

## Author Contributions

All authors listed, have made substantial, direct and intellectual contribution to the work, and approved it for publication.

## Funding

MVS is supported by a project grant from the National Health and Medical Research Council of Australia (1078464). NCR is supported by an early career fellowship from the National Health and Medical Research Council of Australia (1072057).

## Conflict of Interest Statement

The authors declare that the research was conducted in the absence of any commercial or financial relationships that could be construed as a potential conflict of interest. The Review Editor, TK, and handling Editor declared their shared affiliation, and the handling Editor states that the process nevertheless met the standards of a fair and objective review.
